# Mediators of a Physical Activity Intervention on Cognition in Breast Cancer Survivors: Evidence From a Randomized Controlled Trial

**DOI:** 10.2196/13150

**Published:** 2019-10-11

**Authors:** Sheri J Hartman, Lauren S Weiner, Sandahl H Nelson, Loki Natarajan, Ruth E Patterson, Barton W Palmer, Barbara A Parker, Dorothy D Sears

**Affiliations:** 1 Department of Family Medicine and Public Health University of California, San Diego La Jolla, CA United States; 2 UC San Diego Moores Cancer Center University of California, San Diego La Jolla, CA United States; 3 Veterans Affairs San Diego Healthcare System San Diego, CA United States; 4 Department of Psychiatry University of California, San Diego La Jolla, CA United States; 5 Department of Medicine University of California, San Diego La Jolla, CA United States; 6 College of Health Solutions Arizona State University Phoenix, AZ United States

**Keywords:** cognitive function, exercise, anxiety, neoplasms

## Abstract

**Background:**

Emerging research suggests that increasing physical activity can help improve cognition among breast cancer survivors. However, little is known about the mechanism through which physical activity impacts cancer survivors’ cognition.

**Objective:**

The objective of this secondary analysis examined physical and psychological function potentially linking physical activity with changes in cognition among breast cancer survivors in a randomized controlled trial where the exercise arm had greater improvements in cognition than the control arm.

**Methods:**

A total of 87 sedentary breast cancer survivors were randomized to a 12-week physical activity intervention (n=43) or control condition (n=44). Objectively measured processing speed (National Institutes of Health Toolbox Oral Symbol Digit), self-reported cognition (patient-reported outcomes measurement information system [PROMIS] cognitive abilities), PROMIS measures of physical and psychological function (depression, anxiety, fatigue, and physical functioning), and plasma biomarkers (brain-derived neurotrophic factor, homeostatic model assessment 2 of insulin resistance, and C-reactive protein [CRP]) were collected at baseline and 12 weeks. Linear mixed-effects models tested intervention effects on changes in physical and psychological function variables and biomarkers. Bootstrapping was used to assess mediation. Exploratory analyses examined self-reported cognitive abilities and processing speed as mediators of the intervention effect on physical functioning.

**Results:**

Participants in the exercise arm had significantly greater improvements in physical functioning (beta=1.23; 95% CI 2.42 to 0.03; *P*=.049) and reductions in anxiety (beta=−1.50; 95% CI −0.07 to −2.94; *P*=.04) than those in the control arm. Anxiety significantly mediated the intervention effect on cognitive abilities (bootstrap 95% CI −1.96 to −0.06), whereas physical functioning did not (bootstrap 95% CI −1.12 to 0.10). Neither anxiety (bootstrap 95% CI −1.18 to 0.74) nor physical functioning (bootstrap 95% CI −2.34 to 0.15) mediated the intervention effect on processing speed. Of the biomarkers, only CRP had greater changes in the exercise arm than the control arm (beta=.253; 95% CI −0.04 to 0.57; *P*=.09), but CRP was not associated with cognition; therefore, none of the biomarker measures mediated the intervention effect on cognition. Neither cognitive abilities (bootstrap 95% CI −0.06 to 0.68) nor processing speed (bootstrap 95% CI −0.15 to 0.63) mediated the intervention effect on physical function.

**Conclusions:**

Physical activity interventions may improve self-reported cognition by decreasing anxiety. If supported by larger studies, reducing anxiety may be an important target for improving self-reported cognition among cancer survivors.

**Trial Registration:**

ClinicalTrials.gov NCT02332876; https://clinicaltrials.gov/ct2/show/NCT02332876

## Introduction

### Background

The number of breast cancer survivors in the United States is expected to rise dramatically with its aging population and increased rates of breast cancer survival. Cognitive impairment is a disruptive and persistent condition that is common among breast cancer survivors [1]. Breast cancer survivors often show deficits on objective neurocognitive measures and self-reported measures of cognition, which assess different, yet important, aspects of cognition [2]. Cognitive difficulties can impact quality of life and ability to return to work [3]. Therefore, identifying effective interventions to improve cognition is a research priority in cancer survivorship [4].

A potential intervention is physical activity, recommended by the National Comprehensive Cancer Network for cancer-related cognitive dysfunction [4]. Breast cancer survivors often reduce physical activity after treatment and have low levels of physical activity [5]. The low activity levels in breast cancer survivors and the benefits of physical activity for cognition among noncancer populations [6] suggest that physical activity could be an important target for breast cancer survivors. Recent trials, including our 12-week intervention in 87 breast cancer survivors [7], have shown that increasing physical activity improves objective and self-reported cognition in cancer survivors [7-9]. As the intervention literature continues to grow, there is a need to understand the mechanisms driving the effects of physical activity on cognition [9].

A hypothesized mechanism linking physical activity with cognition is improvements in physical and psychological function. Problems with self-reported cognition have consistently been associated with poorer physical function, anxiety, depression, and fatigue, which are often elevated or impaired in cancer survivors [10-12]. Physical activity in cancer survivors can improve all these aspects of physical and psychological function [13-15]; therefore, physical and psychological function may mediate the relationship between physical activity and self-reported cognition. A longitudinal observational study in 1477 breast cancer survivors found that distress and fatigue were on the pathway between physical activity and self-reported cognition [16]. Physical and psychological function factors may also be associated with objective neurocognitive function. Prospective studies in healthy older adults have shown that poor physical function can predict future cognitive impairment [17-19]. A cross-sectional study in 299 breast cancer survivors found that physical activity was related to improved executive function and working memory and that the effect of physical activity on cognition was partially explained by physical activity’s influence on fatigue [20]. To our knowledge, no randomized controlled trials have explored aspects of physical and psychological function as mediators of the effect of physical activity on cognition in cancer survivors. Given the high prevalence of impaired physical and psychological function in breast cancer survivors and the strong connection between cognition and physical activity, improvements in physical and psychological function are plausible mechanisms through which physical activity may improve cognition [21].

Although cognitive and physical function have been shown to be related among cancer survivors [22,23], the direction of this relationship has not been studied. Evidence from cohort studies and randomized controlled trials in older adults suggests that cognitive impairments may precede physical decline [24,25]. Therefore, improvements in cognition may mediate the effects of physical activity on improved physical function.

Physical activity is thought to positively influence cognition via several biological mechanisms including increased brain-derived neurotrophic factor (BDNF), improved metabolic function, and reduced systemic inflammation. BDNF is a biomarker of brain health. BDNF levels are correlated with processing speed [26] and are significantly elevated after aerobic physical activity [27]. In noncancer populations, BDNF mediates the effects of physical activity on neurocognitive outcomes [28] and is positively related to objective [29] and self-reported [30] cognition in cancer patients. BDNF is regulated by energy balance, insulin, and inflammatory cytokines; thus, it is likely part of a central mechanism through which physical activity integrates with elements of metabolism to impact cognition. In fact, measures of metabolic dysfunction, for example, insulin resistance measured by the homeostatic model assessment of insulin resistance (homeostatic model assessment of insulin resistance [HOMA-IR] or homeostatic model assessment 2 of insulin resistance [HOMA2-IR], calculated using fasting plasma insulin and glucose) [31,32], and systemic inflammation, for example, levels of C-reactive protein (CRP), are inversely associated with physical activity [33,34] and cognitive performance [33,35]. Insulin resistance/HOMA-IR is associated with cognitive decline in diabetic [36] and nondiabetic populations [37,38]. Routine physical activity is anti-inflammatory [10] and improves insulin sensitivity [39]. Physical activity interventions in cancer survivors result in beneficial effects on inflammation and insulin pathway biomarkers [39]. Interestingly, exercise frequency and an anti-inflammatory genotype *each* predicted better cognitive performance in a sample of breast cancer survivors [40]. Other accumulating evidence supports a direct link between inflammation and cognition in breast cancer survivors. In breast cancer patients studied before and up to 2 years post treatment, CRP levels were inversely correlated with cognition [41]. Breast cancer survivors have elevated systemic inflammation that is prevalent for decades post treatment and that is associated with cognitive decline [42,43]. In noncancer populations, CRP and insulin resistance have been associated with neurocognitive impairment [44]. Therefore, BDNF, systematic inflammation, and insulin resistance are potential putative mechanisms that could causally link physical activity with cognition in cancer survivors.

### Objectives

The secondary analysis (this study) examined physical and psychological function and biological mechanisms potentially linking physical activity with changes in objective and self-reported cognition among breast cancer survivors enrolled in a 12-week randomized controlled trial. In the primary study of 87 breast cancer survivors, the exercise arm had greater improvements in objectively measured processing speed and self-reported cognitive abilities than the control arm [7]. The primary aim of this analysis was to explore whether proposed physical and psychological function measures (depression, anxiety, fatigue, and physical functioning) and mechanistic biomarkers (BDNF, HOMA2-IR, and CRP) mediated intervention effects on cognition. For the mediation analyses, the following steps were taken: (1) intervention effects on potential mediators (physical/psychological function/biomarkers) were examined, (2) associations between potential mediators (physical /psychological function/ biomarkers) with objectively measured processing speed and self-reported cognitive abilities were examined, and (3) if changes in potential mediators (physical/psychological function/biomarkers) mediated the relationship between the intervention effect on objectively measured processing speed and self-reported cognitive abilities was tested. We a priori hypothesized that, compared with breast cancer survivors in the control arm, participants in the exercise arm would have improvements in physical and psychological function and biomarkers, and that these factors would mediate the relationship between physical activity and cognitive outcomes. To address uncertainties about the directionality of the relationship between physical function and cognition, we also conducted exploratory analyses assessing self-reported cognitive abilities and objectively measured processing speed as mediators of the relationship between physical activity and physical function.

## Methods

### Participants

A total of 87 breast cancer survivors were enrolled in a randomized controlled trial of a 12-week physical activity intervention [21]. Data were collected from February 2015 to July 2016. The study was approved by the University of California (UC) San Diego Institutional Review Board (protocol number 140694), and all participants provided written informed consent. The trial was registered on ClinicalTrials.gov (NCT02332876).

Eligible participants were female breast cancer survivors who were aged 21 to 85 years, who completed breast cancer surgery less than 5 years ago, and who completed chemotherapy or radiation treatment. Other inclusion criteria included the following: self-reporting less than 60 min of moderate-to-vigorous physical activity (MVPA) in 10-min bouts per week; self-reported fogginess or worsening of memory, thinking, or concentration; and internet access. Exclusion criteria included the following: any medical condition that could preclude safe participation in an unsupervised physical activity intervention and other primary or recurrent invasive cancer within the last 10 years.

### Procedures

Detailed information on study procedures and the intervention have been published [21]. Briefly, women were primarily recruited through cancer registries. Potential participants were phone-screened for eligibility and then scheduled for an in-person visit at UC San Diego Moores Cancer Center, during which participants completed questionnaires, a fasting blood draw, and neurocognitive tests. Height and weight were measured. Next, participants received an ActiGraph GT3X+ accelerometer (ActiGraph, LLC.) to wear for 7 days and return at the randomization visit. At the randomization visit, 87 breast cancer survivors were randomized in a 1:1 allocation ratio to receive either a 12-week physical activity intervention (exercise arm, n=43) or waitlist wellness contact control condition (control arm, n=44). All baseline measures were repeated at 12 weeks. A participant from each arm was lost to follow-up, resulting in 98% retention (exercise, 42/43 and control, 43/44) [7].

#### Physical Activity Intervention (Exercise Arm)

Participants randomized to the exercise arm completed an in-person visit that lasted 30 min to 45 min. At this visit, participants were first taken on a 10-min walk to learn what moderate-intensity activity felt like. Then, using motivational interviewing techniques, the interventionist helped the participant set a personalized physical activity goal. Participants could set any starting goal they wanted and were encouraged to gradually increase aerobic exercise to meet the study goal of at least 150 min of MVPA per week [45]. To support self-monitoring and accountability, they received a Fitbit One and were informed that the interventionist could see the Fitbit data and would be checking on their activity weekly. Interventionists provided feedback on Fitbit data during 2 scheduled phone calls at the 2- and 6-week time points and as needed. Participants also received emails every 3 days throughout the intervention with theory-based content and reminders to wear and sync their Fitbit.

#### Waitlist Wellness Contact Control Condition (Control Arm)

Participants randomized to the control arm received emails on the same schedule as the exercise arm. Emails focused on various women’s health topics of interest to breast cancer survivors including general brain health, healthy eating, and reading nutrition labels. Content of the emails were specifically chosen to be topics of interest but were very brief and strictly informational to not encourage behavior change. After completing the 12-week measures, participants in the control arm received the physical activity intervention described above.

### Measures

#### Physical and Psychological Function

Anxiety, depression, fatigue, and physical function were measured using patient-reported outcomes measurement information system (PROMIS) measures developed by the National Institutes of Health (NIH) for cancer survivors. Using a computer adaptive format, questions assessed symptoms over the past 7 days. Higher scores on the physical functioning measure indicate better functioning. Higher scores on all other measures indicate worse functioning. Raw scores for each PROMIS measure are reported on a standardized T-score metric (mean 50, SD 10), separately for each measure [46]. PROMIS measures have undergone rigorous evaluation and validation in cancer survivors and have shown responsiveness to both improvements and declines in symptoms and function over time, as well as sensitivity to detect differences between groups for which a change is expected versus comparison groups for which no change is expected [47]. In line with previous studies in cancer survivors, a clinically meaningful difference was defined as a 3-point difference in scores [46,48].

#### Biomarkers

Biomarkers of interest were BDNF, CRP, and HOMA2-IR. In total, 12 mL of fasting blood was collected in EDTA tubes; plasma was immediately isolated by centrifugation at 4°C, then aliquoted and stored at −80°C. Biomarkers were assayed after all data collection was completed to minimize batch-to-batch variation. Paired samples were run side by side, and quality control and normalization control samples were included in all assay runs. Glucose concentrations were measured using a standard glucose oxidase method (YSI 2900 Biochemistry Analyzer). Plasma BDNF, CRP, and insulin concentrations were determined using high-sensitivity immunoassays (Meso Scale Discovery: custom kit [BDNF], catalog number K15198D [CRP], and catalog number K15164C, [insulin]) run at the NIH-funded UC San Diego Clinical and Translational Research Institute Biomarker Laboratory. BDNF concentrations for individual samples were normalized to the normalization control sample set run in quadruplicate on each of the 3 assay plates. Coefficients of variance were 6.1% (BDNF), 2.9% (CRP), 7.1% (glucose), and 4.6% (insulin). HOMA2-IR was calculated using fasting glucose and insulin concentrations [49] using the publicly downloadable HOMA2 calculator [50]. HOMA2-IR is a model-derived estimate of insulin resistance that uses a more sophisticated calculation than the linear equation for HOMA-IR.

#### Cognitive Functioning

Processing speed was measured with the Oral Symbol Digit test from the NIH Toolbox Cognition Domain [51]. This computer-based version of the Wechsler Adult Intelligence Scale Digit-Symbol-Coding test has been validated and normed in individuals aged 3 to 85 years. The test provides a raw score (possible range: 0-144), with higher scores indicating better processing speed [51].

Self-reported cognition was measured with the PROMIS cognitive abilities questionnaire that assesses patient-perceived functional abilities in the past 7 days. Higher scores on the cognitive abilities measure indicate more positive perceptions of cognition. This measure provides standardized T-scores that have demonstrated good reliability and validity with previous measures including the functional assessment of cancer therapy-cognitive function measure [52].

#### Physical Activity

Change in MVPA was measured with the ActiGraph GT3X+. Wear time was screened for in Actilife software (ActiGraph) using the guidelines by Choi et al [53]. Sufficient wear time was defined as 5 days with more than or equal to 600 min or 3000 min across 4 days. Time spent in minutes of MVPA was derived using the 1952 cut point [54]. The ActiGraph has been validated against heart rate telemetry and total energy expenditure [55].

#### Demographic and Clinical Variables

Self-reported demographics, including age, education, income, race/ethnicity, and marital status, were collected at baseline. Body mass index (BMI) was calculated from height and weight measurements collected at baseline. Breast cancer information and treatment details were obtained from medical charts.

### Statistical Analysis

Unless otherwise specified, all analyses were conducted using SAS version 9.4 (SAS Institute, Inc). All analyses were performed using an intent-to-treat principle. Longitudinal random-effects models were developed. This method uses all available data, does not omit subjects with missing data, and provides unbiased results provided the data conform to a *missing at random* missing data mechanism. Group differences in baseline characteristics were assessed using *t* tests and chi-square tests. Mixed-effects regression models with a subject-level random intercept and an unstructured covariance structure, as determined by model Akaike Information Criterion comparisons in the main effects models, were used for all other models. Models assessing intervention effects on repeated measures (at baseline and 12 weeks) of anxiety, depression, fatigue, physical function, and biomarkers (BDNF, HOMA2-IR, and CRP) included fixed effect terms for group, time point (baseline or 12 weeks), and time-by-group interaction. Contrasts were used to calculate the difference of change based on the regression model when examining group differences. Each outcome was assessed in a separate model. All biomarkers were log transformed before analyses to correct for right-skewed residuals.

Assuming a 2-sided test with an alpha of .05 and a sample size of 80 with 1:1 randomization to treatment or control, there was 80% power to detect a main effect of 0.32.

#### Mediation Analysis

Owing to the small sample size, we chose an a priori significance level of *P*<.10 to assess mediation. Mediation analyses were based on the approach of Baron and Kenny [56]. This approach states that the following conditions must be met for a test of potential mediation: (1) the independent variable (group) has a significant effect on the mediator (physical/psychological function/biomarker)—path *a*, (2) the mediator (physical/psychological function/biomarker) is associated with the outcome variable (cognition)—path *b*, and (3) the independent variable (group) has a significant effect on the outcome variable (cognition)—path *c.* If these conditions are met, a final analysis involves a multivariable model of the independent variable (group) predicting change in the outcome variable (cognition), controlling for the mediator (physical/psychological function/biomarker)—path *c*’. Path c (intervention effect on cognition) was previously published [7], and only 2 of the cognition measures, processing speed and self-reported cognitive abilities, met our a priori significance level to be included in this mediation analyses.

In the mediation analysis, a drop in predictive power from path *c* to path *c’* (the indirect effect) suggests the existence of a mediation effect. The significance of this drop in predictive power was conducted using bootstrapping, whereby the indirect effect (*c-c’*) was generated 200 times, and this sampling distribution was used to determine bounds for the 95% CI of the indirect effect. Bootstrapping was performed in R [57,58]. As we had a repeated measured design, we used the lme4 package [59] to apply the repeated measures mixed-effects model to all steps in the mediation analysis. Mediation analysis was conducted for all physical or psychological function or biomarker variables found to have a significant path *a*, *b*, and *c* at the *P*<.10 level. Intervention effects on cognition were reported in the main outcomes analysis for the study [7]. The main effects of group on cognition were determined similar to our main effects model described above. A mixed-effects regression model of each cognition variable of interest included fixed effect terms for group, time point (baseline or 12 weeks), and time-by-group interaction [7].

Exploratory analyses testing self-reported cognitive abilities and objectively measured processing speed as mediators of the intervention effect on physical function used the same mediation models and bootstrapping procedure described above.

## Results

### User Statistics

Participants (n=87) were, on average, aged 57 (SD 10.4) years and 30 (SD 16.7) months post breast cancer surgery. Majority of the participants were diagnosed with stage 1 disease (61%, 53/87), received chemotherapy (53%, 46/87), and were taking an aromatase inhibitor or tamoxifen (70%, 61/87) at the time of study enrollment. Mean T-scores on the physical and psychological function measures ranged from a mean of 50.4 (SD 7.98) for depression to a mean of 55.2 (SD 7.72) for anxiety across the study arms. See Table 1 for baseline characteristics stratified by study arm; there were no significant differences between the exercise and control arms in baseline characteristics (*P*>.05).

**Table 1:** Baseline characteristics by study arm among breast cancer survivors enrolled in a randomized trial of physical activity (N=87).

Baseline characteristics			Exercise intervention (n=43)	Wellness control (n=44)	*P* value
**Demographics^a^**					
	Age (years), mean (SD)		58.2 (11.37)	56.2 (9.30)	.35
	**Education, n (%)**				.69
		Some college or less	14 (33)	11 (25)	—^b^
		College graduate	18 (42)	22 (50)	—
		Master’s degree or higher	11 (26)	11 (25)	—
	Married or living with partner, n (%)		32 (76)	31 (71)	.68
	**Ethnicity, n (%)**				.74
		Not Hispanic or Latino	35 (81)	37 (84)	—
		Hispanic or Latino	8 (19)	7 (16)	—
	**Race, n (%)**				.62
		White	36 (84)	35 (80)	—
		Nonwhite	7 (16)	10 (23)	—
Body mass index (kg/m^2^), mean (SD)			26.7 (6.20)	27.3 (6.40)	.63
Time since breast cancer surgery (months), mean (SD)			30.3 (17.41)	30.0 (16.08)	.99
**Cancer stage, n (%)**					.79
	Stage 1		27 (63)	26 (59)	—
	Stage 2		12 (28)	15 (34)	—
	Stage 3		4 (9)	3 (7)	—
Received chemotherapy, n (%)			23 (54)	23 (52)	.91
Current aromatase inhibitor or tamoxifen, n (%)			31 (72)	30 (68)	.69
**Physical and psychological functioning, mean (SD)**					
	Physical functioning		50.2 (7.49)	51.8 (6.84)	.32
	Anxiety		54.8 (8.51)	55.6 (6.95)	.65
	Depression		50.0 (8.16)	50.7 (7.88)	.68
	Fatigue		52.5 (7.64)	54.4 (9.17)	.29
**Biomarkers^c^, mean (SD)**					
	Brain-derived neurotropic factor, normalized values		0.3 (0.19)	0.3 (0.29)	.32
	Homeostatic model assessment 2 of insulin resistance		1.2 (0.98)	1.4 (1.12)	.58
	Log C-reactive protein (pg/mL)		14.3 (1.29)	14.6 (1.32)	.31
**Cognition^a^, mean (SD)**					
	Neurocognitive testing: processing speed (raw score)		73.7 (14.55)	77.7 (13.23)	.19
	Self-reported cognitive abilities (T-score)		46.6 (5.96)	44.3 (5.27)	.06

^a^A secondary analysis of the mechanisms underlying the effect of an intervention on cognition is displayed here. The main effects of the intervention on cognition were previously published in a study by Hartman et al [7]

^b^Not applicable.

^c^Biomarker values were log transformed before analysis to correct for right-skewed residuals.

### Main Effects

The overall effects of the intervention on physical activity and cognitive outcomes have been published [7]. Briefly, the exercise arm had significantly greater increases in accelerometer-measured MVPA (mean min/day increase: 14.2 vs −0.7; beta=7.24; *P*<.001) than the control arm from baseline to 12 weeks. Participants in the exercise arm showed significantly greater improvements in objectively measured processing speed than those in the control arm (mean increase score 7.0, SD 10.2 vs 3.0, SD 8.2; beta=2.01; *P*<.05). The between-group difference in self-reported cognitive abilities (beta=.92; *P*=.09) was not statistically significant, but the magnitude of the change within the exercise arm was suggestive of clinically meaningful improvement (average 2.7-point improvement from baseline to 12 weeks) [46].

### Physical and Psychological Function Mediation

**Figure 1:**
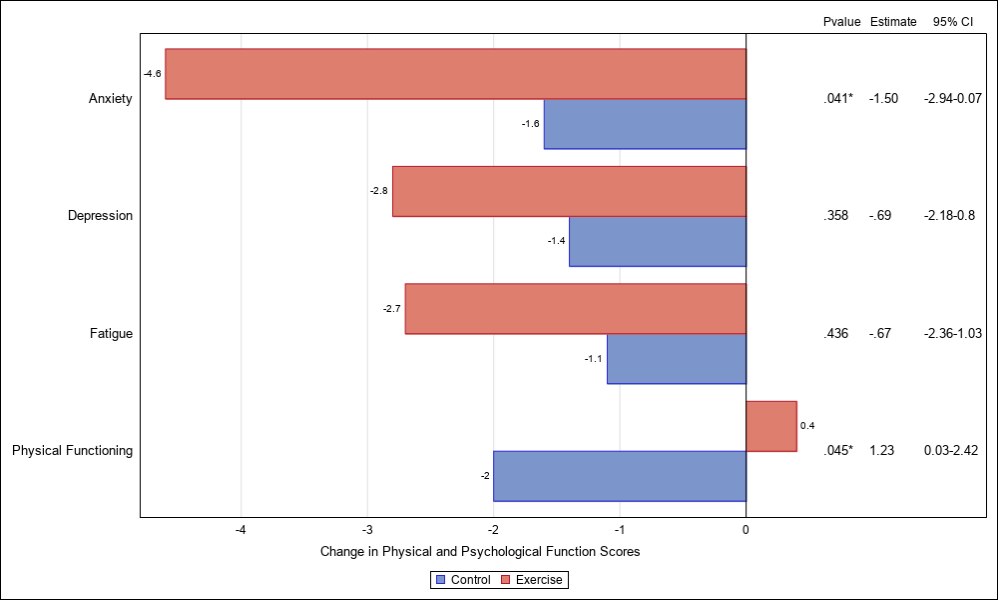
Differences in change from baseline to 12 weeks on measures of physical and psychological function by randomization group among breast cancer survivors enrolled in a randomized controlled trial of physical activity (N=87). Estimate: estimate of difference between groups for change in quality of life scores.

**Figure 2:**
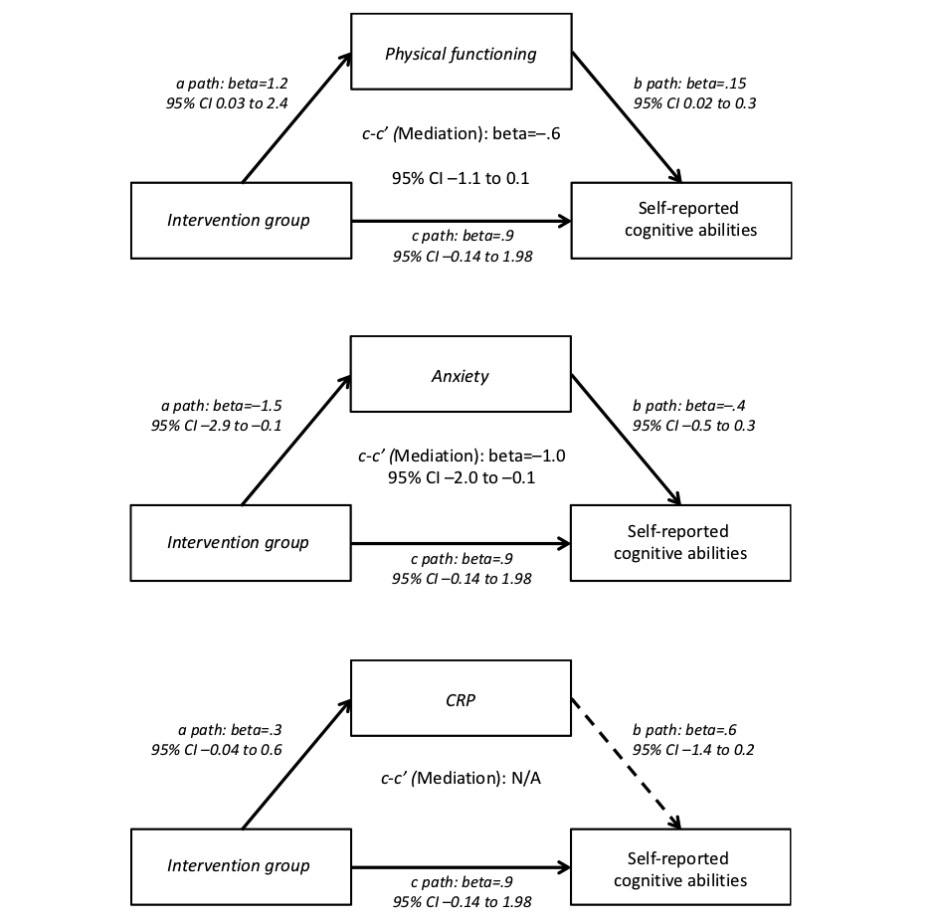
Bootstrap mediation analysis of anxiety, physical functioning, and C-reactive protein self-reported cognitive abilities among breast cancer survivors enrolled in a randomized trial of physical activity (N=87). Solid arrow lines indicate a *P* value less than .10. Dashed arrow lines indicate a *P* value greater than or equal to .10.

**Figure 3:**
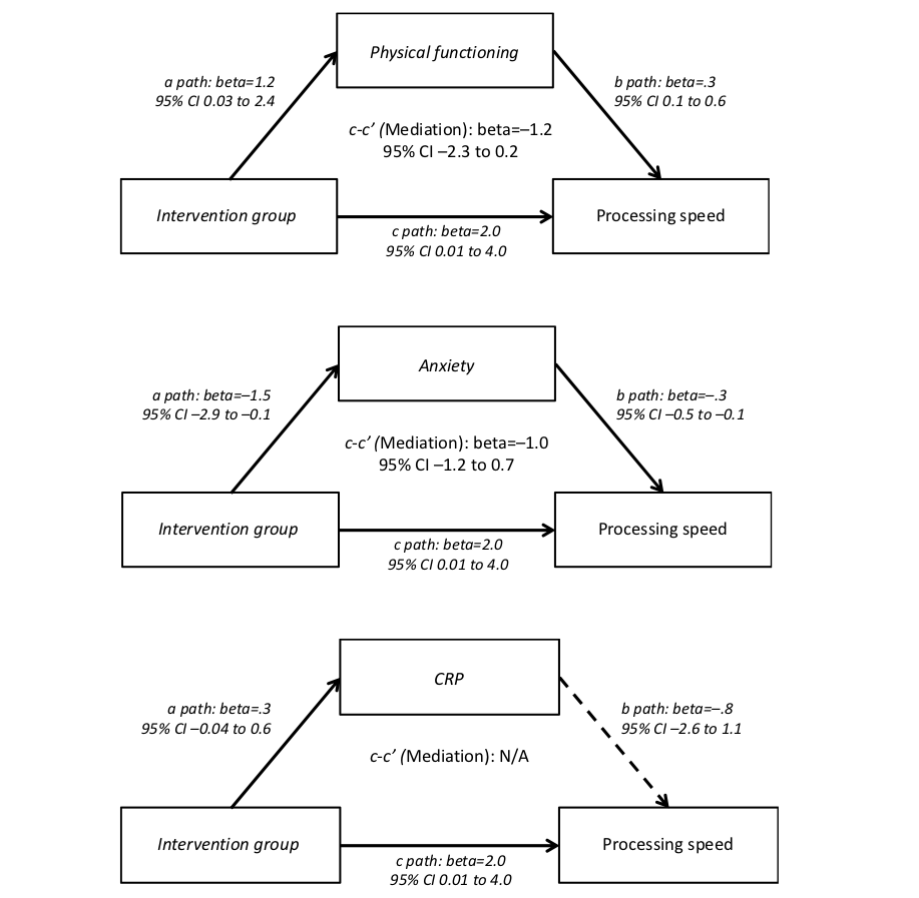
Bootstrap mediation analysis of anxiety, physical functioning, and C-reactive protein on processing speed among breast cancer survivors enrolled in a randomized trial of physical activity (N=87). Solid arrow lines indicate a *P* value less than .10. Dashed arrow lines indicate a *P* value greater than or equal to .10.

Changes in physical and psychological function measures from baseline to 12 weeks in the exercise versus control arms are presented in Figure 1. The exercise arm had significantly greater improvements in physical functioning than the control arm (beta=1.23; 95% CI −0.42 to 0.03; *P*=.049). The exercise arm also showed significantly greater reductions in anxiety than the control arm (beta=−1.50; 95% CI −0.07 to −2.94; *P*=.04). Furthermore, changes in each of physical functioning and anxiety were significantly associated with the change in cognition (Figures 2 and 3 and Multimedia Appendix 1). Therefore, conditions were met to test for the potential mediation effects of anxiety and physical functioning. Results indicate that anxiety significantly mediated the intervention effect on cognitive abilities (see Figure 2). Differences between the exercise and control arms in changes in cognitive abilities were, in part, because of greater decreases in anxiety among intervention participants compared with those in the control group (bootstrap 95% CI −1.96 to −0.06). Physical functioning did not mediate the intervention effect on cognitive abilities (bootstrap 95% CI −1.12 to 0.10). Neither anxiety (bootstrap 95% CI −1.18 to 0.74) nor physical functioning (bootstrap 95% CI −2.34 to 0.15) mediated the effect of the intervention on processing speed (see Figure 3). Exploratory analyses found that neither cognitive abilities (bootstrap 95% CI −0.06 to 0.68) nor processing speed (bootstrap 95% CI −0.15 to 0.63) mediated the intervention effect on physical function.

### Biomarker Mediation

The exercise arm had greater reductions in CRP than the control arm (beta=.253; 95% CI −0.04 to 0.57; *P*=.09; see path *a* in Figures 2 and 3 and Multimedia Appendix 1). Change in CRP was not associated with either measure of cognition (see path *b* in Figures 2 and 3 and Multimedia Appendix 1); therefore, mediation could not be tested. There were no between-group differences in changes in BDNF (beta=.092; 95% CI −0.25 to 0.43; *P*=.59) or HOMA2-IR (beta=.05; 95% CI −0.12 to 0.22; *P*=.55); therefore, conditions were not met to test for mediation, and no further analyses were conducted.

## Discussion

### Principal Findings

This is the first known published randomized trial with breast cancer survivors to explore potential physical and psychological function and biological mechanisms underlying the effect of a physical activity intervention on objective and self-reported cognition. Results indicated that reductions in anxiety may have contributed to improvements in self-reported cognitive abilities, but there was no evidence of mediation for objectively measured processing speed or other measures of physical or psychological function. In addition, there was no evidence that the proposed biological mechanisms mediated the intervention effect on objective or self-reported cognition.

### Comparison With Previous Studies

Findings from this pilot study support the importance of anxiety for self-reported cognition and the potential for physical activity interventions to reduce anxiety in breast cancer survivors. Although previous physical activity intervention trials with cancer survivors that have examined physical or psychological function factors as possible mechanisms by which physical activity impacts cognition could not be identified, this finding is consistent with the published literature suggesting that self-reported cognition is likely a psychosocial aspect of cancer and indicator of psychological distress [60,61]. Self-reported cognition is consistently associated with other dimensions of psychological functioning such as depression and anxiety [2,61,62] that are elevated in breast cancer survivors and have been shown to improve with physical activity [14,63]. These results are also consistent with the notion that self-reported cognition is a distinct construct from objective neurocognitive function [2,64] and that there may be different mechanisms of the effects of physical activity on self-reported and objectively measured cognition.

Contrary to expectations, anxiety was the only physical or psychological function factor that was a significant mediator of the intervention effects on cognition. This finding may be because of the general lack of problems with physical and psychological function in our sample at baseline. It must be noted that, among all physical and psychological function factors examined, anxiety had the highest average scores at baseline (indicating higher levels of anxiety) and was the only variable to achieve a clinically meaningful change, which has been defined as a 3-point difference in scores for these measures [46]. Exploratory analyses to better understand the direction of the relationship between physical function and cognition were also nonsignificant. Research from the literature on aging suggests that both cognitive impairments precede physical declines [24,25], and physical declines precede cognitive declines [17,19]. However, this sample did not report impaired physical function at baseline, which might have limited the potential for physical functioning to mediate the relationship between physical activity and cognition or for cognition to mediate improvements in physical function. Fully powered trials, and trials targeting women with lower physical functioning, should be conducted to determine whether physical activity–associated improvements in day-to-day physical functioning are part of the causal pathway through which exercise improves cognition. Overall, this study provides preliminary evidence that in breast cancer survivors, physical activity interventions may improve self-reported cognition by reducing anxiety. Future studies with longer time frames and more distressed participants may be able to elicit clinically meaningful benefits to other aspects of physical and psychological functioning. Future research should also explore other aspects of physical and psychological function as potential mechanisms underlying the impact of physical activity interventions on cognition.

Although the intervention showed some reduction in systemic inflammation, as measured by CRP, this was not associated with cognition. Contrary to expectations, the intervention did not improve BDNF or HOMA2-IR. These findings may be because of having a sample with a normal to overweight BMI and, on average, low HOMA2-IR, small sample size, and the short intervention duration. Previous larger physical activity randomized controlled trials with breast cancer survivors have found that the effects of physical activity on the insulin pathway are stronger among obese survivors [39]. BDNF has been understudied in breast cancer survivors; however, a review of randomized controlled trials of physical activity interventions in noncancer populations found improvements in BDNF in 3 out of 5 trials, with 2 of the trials being longer than 6 months [27]. Findings from this study suggest that among breast cancer survivors with a generally healthy BMI, neurotrophic factors, inflammation, or insulin pathways may not be the mechanisms for changes in cognition in a physical activity intervention. However, fully powered trials are needed to confirm these results.

As the research testing the impact of physical activity on cancer-related cognitive impairments grows, it is important to continue exploring the mechanisms that causally link physical activity and cognition. Future studies should consider factors related to biological and cellular aging. It has been hypothesized that MVPA can slow, or even reverse, cellular aging and thereby improve cognitive performance at the cellular level. This association is particularly relevant for cancer survivors who may experience accelerated aging caused by chemotherapy and psychological stress [1,65]. Other potential mechanisms may be related to the impact of physical activity on brain structure and function. Neuroimaging studies in breast cancer survivors have found structural changes including reduction in gray matter volume following chemotherapy, which has been associated with impairments in processing speed [66,67]. Although the direct impact of increasing physical activity on brain volume has not been tested, there is some evidence that hippocampal volumes are larger in breast cancer survivors with high cardiovascular fitness levels [68]. Physical activity also has the potential to increase cerebral blood flow, which may slow neurodegeneration [69]. Exploring whether the rate of cellular aging can be slowed by physical activity and whether physical activity can improve brain structure and function are important directions for future research with cancer survivors.

### Limitations and Strengths

The small sample size may not have provided sufficient power to detect significant mediation, and only bivariate mediation possibilities were considered. Subsequent studies with larger samples should consider multiple mediating mechanisms. Nonetheless, information gathered from these analyses can help generate new hypotheses about mechanisms and guide future studies to improve cognitive function in breast cancer survivors. The short duration and relative physical and mental health of the study population may also have limited our ability to detect mediation in the chosen mechanisms. Future studies with longer durations and more diverse populations that incorporate novel measures of cellular aging and neuroimaging are needed to continue advancing the field. Owing to the pilot nature of this study with limited power, we also did not adjust for multiple comparisons. In addition, these results are limited to a single measure of processing speed, and the findings cannot be generalized to other processing speed measures or aspects of cognition, such as memory, attention, and executive function, which are often impacted by cancer and its treatments [70]. Another limitation is that the PROMIS measures may not have been sufficiently sensitive to the magnitude of change achieved in this study. A longitudinal study of almost 3000 cancer survivors demonstrated that effect sizes of 0.24 to 0.71 were needed to detect declines and improvements across different PROMIS measures [47].

Despite these limitations, the study had many strengths including the randomized design and objective measurement of physical activity. In addition, this study uniquely focused on physical and psychological function and biological mechanisms of action across both objective and self-report measures of cognition. Furthermore, bootstrap methods to assess mediation were used, which is recommended in small to moderate samples [71]. This research extends what is known about the impact of physical activity on cognitive function to examine mechanisms of change. Identifying underlying mechanisms is critical for determining modifiable intervention targets and enhancing intervention efficacy.

### Conclusions

Results of this novel study provide preliminary evidence that an intervention that increases physical activity may improve self-reported cognition by decreasing anxiety. If supported by larger studies, recommending increasing physical activity may be an effective strategy for reducing anxiety and improving self-reported cognition among cancer survivors. Continued research in this area is needed to determine mechanisms of change for objectively measured cognition.

## Appendix

Multimedia Appendix 1 Bootstrap mediation analysis of anxiety, physical functioning, and C-reactive protein on processing speed and self-reported cognitive abilities among breast cancer survivors enrolled in a randomized trial of physical activity. (N=87).

## Abbreviations

BDNF: brain-derived neurotrophic factor
BMI: body mass index
CRP: C-reactive protein
HOMA2-IR: homeostatic model assessment 2 of insulin resistance
HOMA-IR: homeostatic model assessment of insulin resistance
MVPA: moderate-to-vigorous physical activity
NCI: National Cancer Institute
NIH: National Institutes of Health
PROMIS: patient-reported outcomes measurement information system
UC: University of California
