# Supplementary material for: Mediators of a Physical Activity Intervention on Cognition in Breast Cancer Survivors: Evidence From a Randomized Controlled Trial
Source: JMIR Cancer. 2019 Oct 11;5(2):e13150. doi: 10.2196/13150 (PMC6914286; doi:10.2196/13150)
Supplement: Multimedia Appendix 1 [file cancer_v5i2e13150_app1.pdf]

Supplemental Table 1. Bootstrap mediation analysis of anxiety, physical functioning, and CRP on processing speed and self-reported cognitive abilities among breast cancer survivors enrolled in a randomized trial of physical activity. (N=87)

		a (Intv on mediator)				***b (mediator on cognition)***				***c (total effect, Intv on cognition)***				***c' (direct effect, Intv on cognition, controlling for mediator)***				***c-c' (indirect effect)***		
		a				***b***				***c***		*95% CI*		***c'***		*95% CI*		***Mediation***		
Cognition	*Mediators*	*Coeff*	*p-value*	*95% CI*		*Coeff*	*p-value*	*95% CI*		*Coeff*	*p-value*			*Coeff*	*p-value*			*Coeff*	*95% CI*	
**Processing speed**	Anxiety	-1.5	0.04	-2.94	-0.07	-0.3	0.02	-0.53	-0.06	2.0	0.049	0.01	4.01	1.86	0.07	-0.17	3.90	-1.0	-1.2	0.7
	Physical functioning	1.2	0.05	0.03	2.42	0.3	0.02	0.06	0.63					1.57	0.12	-0.43	3.58	-1.2	-2.3	0.2
	CRP	0.3	0.09	-0.04	0.57	-0.8	0.42	-2.60	1.10					N/A		N/A		N/A		
**Self-reported cognitive abilities**	Anxiety	-1.5	0.04	-2.94	-0.07	-0.35	<.01	-0.45	-0.25	0.9	0.087	-0.14	1.98	0.45	0.38	-0.56	1.47	-1.0	-2.0	-0.1
	Physical functioning	1.2	0.05	0.03	2.42	0.15	0.03	0.02	0.27					0.73	0.17	-0.31	1.77	-0.6	-1.1	0.1
	CRP	0.3	0.09	-0.04	0.57	0.6	0.14	-1.39	0.19					N/A		N/A		N/A		
